# *CryoQSeg*: robust particle segmentation for cryo-EM with Qwen-Image-Edit

**DOI:** 10.1107/S2052252526009887

**Published:** 2026-10-15

**Authors:** Yunhai Sun, Jiahao Zhao, Nan Xu, Lei Wang, Wei Ding, Ming Li

**Affiliations:** a Laboratory of Soft Matter Physics, Institute of Physics Chinese Academy of Sciences Beijing 100190 People’s Republic of China; b School of Physical Sciences University of Chinese Academy of Sciences Beijing 100049 People’s Republic of China; c Institute of Automation Chinese Academy of Sciences Beijing 100190 People’s Republic of China; d ScienceOne Team, Beijing, 100190, People’s Republic of China; Boston University School of Medicine, USA

**Keywords:** cryo-EM, protein particle picking, machine learning, visual large model, 3D reconstruction and image processing, automation

## Abstract

Accurate particle picking remains a critical step in cryo-electron microscopy (cryo-EM) single-particle analysis, and the field has witnessed the development of a broad spectrum of approaches – from classical template-based methods to modern deep learning frameworks – to address this task. Here we present *CryoQSeg*, a particle segmentation and picking framework that represents a paradigm shift toward leveraging a state-of-the-art visual large model. Built upon Qwen-Image-Edit-2511 as its core backbone, *CryoQSeg* is fine-tuned through supervised fine-tuning and reinforcement learning on the public CryoPPP benchmark dataset, enabling the generation of high-fidelity, pixel-level particle masks directly from raw micrographs. To translate these masks into precise particle coordinates, we further integrate a hybrid post-processing pipeline that couples Hough circle detection with the segment anything model, forming a seamless end-to-end workflow from raw image input to accurate localization. We evaluate *CryoQSeg* on five representative benchmark datasets, demonstrating competitive performance across a range of protein types and imaging conditions in comparison to mainstream methods including *Topaz* and *CryoSegNet*. Furthermore, we validate the framework on the heterogeneous Crt-SPARTA-gRNA-tDNA complex, where it successfully resolves three distinct oligomeric states – monomer, dimer and tetramer – at high resolutions, despite using a fraction of the micrographs employed in the original study. By bridging modern visual large models with classical and foundation-model-based post-processing strategies, *CryoQSeg* demonstrates the potential of this emerging paradigm to deliver a robust and automated solution for cryo-EM particle picking, thereby contributing to a solid foundation for high-resolution three-dimensional structural determination of biological macromolecules.

## Abstract 2

*CryoQSeg*, a novel cryo-EM particle segmentation algorithm harnessing the state-of-the-art Qwen-Image-Edit visual large model fine-tuned on the CryoPPP dataset, demonstrates outstanding particle picking and segmentation performance for cryo-EM imaging data.

## 1. Introduction

Single-particle cryo-electron microscopy (cryo-EM) (Chari & Stark, 2023 ▸) has become a cornerstone technique in structural biology, enabling high-resolution three-dimensional (3D) reconstruction of biological macromolecules in near-native states. A key step in the cryo-EM workflow is the accurate identification and localization of protein particle projections from raw micrographs, as the quality and completeness of particle selection directly influence the resolution and reliability of subsequent 3D structural reconstruction (Scherer *et al.*, 2014 ▸).

Over the past decades, considerable efforts have been devoted to developing automated particle picking methods (Dhakal *et al.*, 2025 ▸). Early approaches include template-based matching (Wu *et al.*, 2019 ▸) and difference-of-Gaussians (DoG) detection (Pei *et al.*, 2016 ▸), which rely on predefined templates or handcrafted features. More recently, deep-learning-based methods have gained widespread adoption. For instance, *crYOLO* (Wagner *et al.*, 2019 ▸) adapts the YOLO object detection architecture (Jiang *et al.*, 2022 ▸) for rapid particle localization; *Topaz* (Bepler *et al.*, 2019 ▸) employs positive-unlabeled learning to balance sensitivity and specificity; *DeepPicker* (Wang *et al.*, 2016 ▸) utilizes convolutional neural networks to learn particle features from labeled data; and *CryoSegNet* (Gyawali *et al.*, 2024 ▸) combines an attention-gated U-Net (Azad *et al.*, 2024 ▸) with the segment anything model (SAM) (Leng *et al.*, 2024 ▸) to refine particle boundaries. These methods, together with integrated platforms such as *RELION* (Scheres, 2012 ▸) and *CryoSPARC* (Punjani *et al.*, 2017 ▸), have significantly advanced the field by providing automated or semi-automated picking capabilities across a range of cryo-EM datasets. Despite these advances, challenges remain in achieving robust generalization across diverse sample types and imaging conditions, as many methods require dataset-specific parameter tuning or retraining to maintain optimal performance.

Recent advancements in visual large models (VLMs) have demonstrated strong capabilities in computer vision tasks, offering potential to address generalization challenges in cryo-EM particle picking. VLMs, pre-trained on large and diverse image datasets, exhibit enhanced semantic understanding and transferability. However, their direct application to the specialized domain of cryo-EM – with its unique noise characteristics, low signal-to-noise ratios and small object sizes – remains largely unexplored.

In this work, we explore the application of a state-of-the-art visual large model – Qwen-Image-Edit-2511 (Yang *et al.*, 2026 ▸) – to cryo-EM particle segmentation and picking. We adapt this general-purpose model to the cryo-EM domain through supervised fine-tuning and reinforcement learning on the CryoPPP (Dhakal *et al.*, 2023 ▸) benchmark dataset. To translate the resulting pixel-level masks into precise particle coordinates, we further design a hybrid post-processing pipeline that combines Hough circle detection (Seo & Kim, 2015 ▸) with SAM. This integrated framework, which we name *CryoQSeg*, represents a step toward harnessing the capabilities of modern visual large models for cryo-EM particle picking.

**Figure 1:**
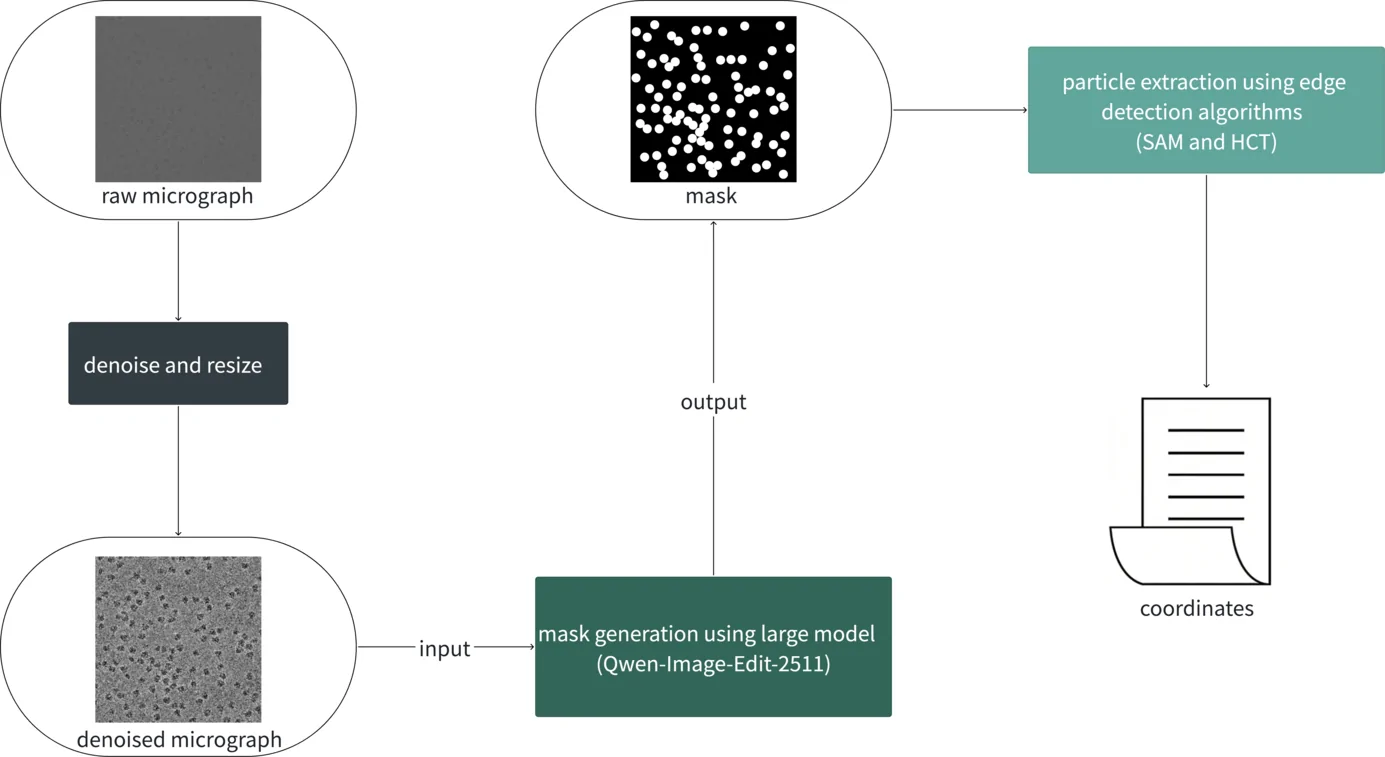
Simplified schematic diagram of the *CryoQSeg* workflow.

We evaluate *CryoQSeg* on a diverse set of five benchmark datasets from the CryoPPP collection, covering a range of protein types and imaging conditions. Additionally, we validate the framework on the Crt-SPARTA-gRNA-tDNA complex, which contains three distinct oligomeric states (monomer, dimer and tetramer) and serves as a challenging test case for generalization across particle sizes and conformations. By exploring the synergy between visual large models and classical computer vision techniques, this work aims to contribute a new methodological perspective to the cryo-EM community and to support downstream high-resolution 3D structural determination. The simplified workflow is illustrated in Fig. 1 ▸.

## 2. Methods

### 2.1. Dataset acquisition

*CryoQSeg* was developed and evaluated using the CryoPPP dataset, a curated resource derived from the Electron Microscopy Public Image Archive (EMPIAR) (Iudin *et al.*, 2023 ▸). This dataset comprises 27 EMPIAR entries spanning diverse protein categories, including membrane proteins, viral capsids, ribosomal complexes and soluble enzymes. For model fine-tuning, 22 entries were used, with all micrographs and corresponding expert-annotated particle masks employed for supervised fine-tuning (Shen, 2024 ▸) and reinforcement learning. The remaining 5 entries, not used during fine-tuning, served as an independent test set to assess generalization.

### 2.2. Input and preprocessing

**Figure 2:**
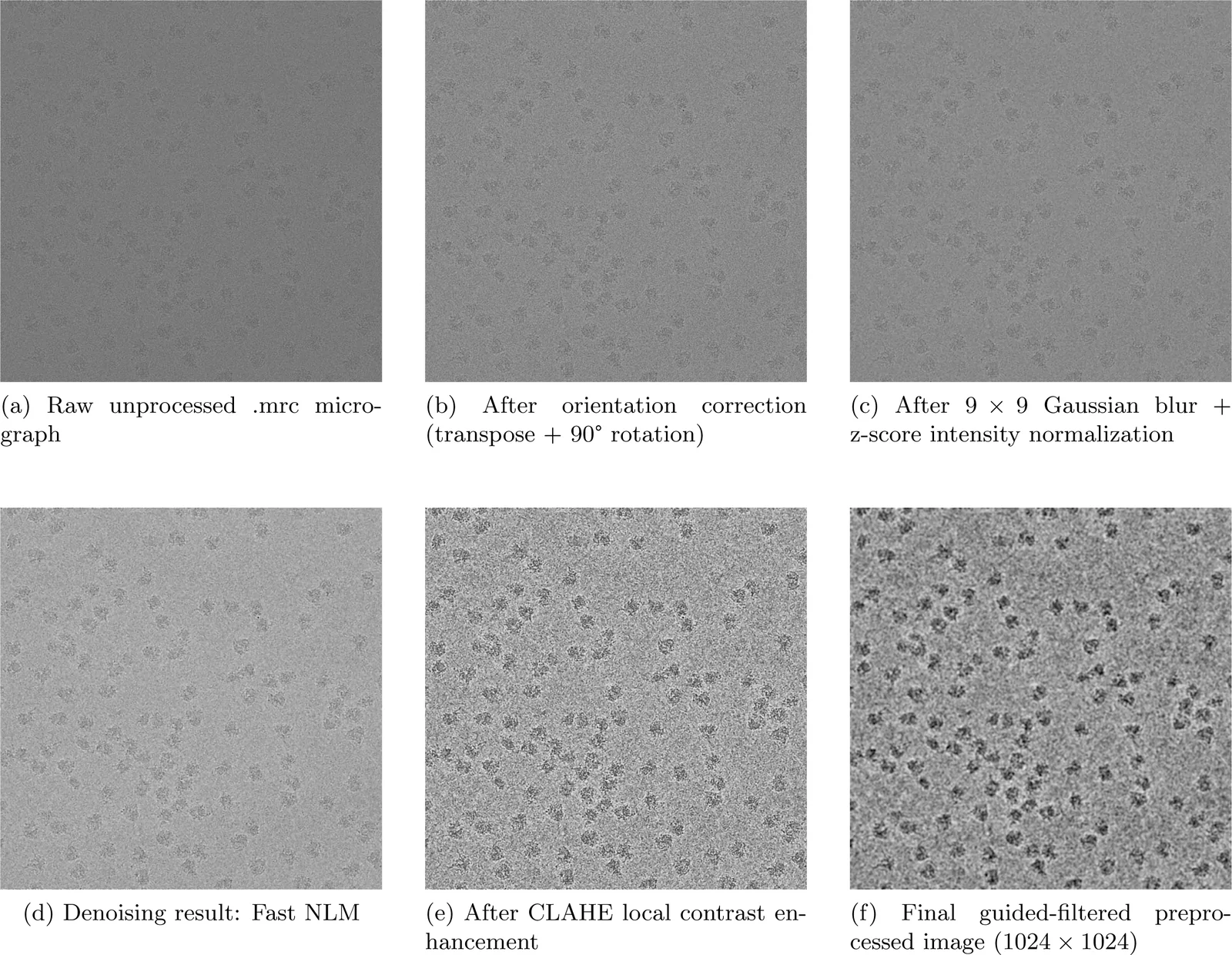
(*a*) Original raw cryo-EM micrograph loaded from .mrc file. (*b*) Micrograph after transposition and 90° rotation for orientation rectification. (*c*) Normalized image after Gaussian smoothing and z-score intensity scaling to 0–255. (*d*) Denoised output combining fast non-local means (NLM) and Wiener filtering with 9 × 9 Gaussian kernel. (*e*) Contrast-adjusted image processed by *CLAHE*. (*f*) Final preprocessed image after guided filtering (Wiener result as guidance map), resized to 1024 × 1024 for model input.

To standardize input characteristics for the visual large model, raw cryo-EM micrographs stored in .mrc format are processed via a fixed multi-step preprocessing pipeline before feeding into Qwen-Image-Edit-2511 (Gyawali *et al.*, 2024 ▸). All intermediate outputs after each processing stage are visualized in Fig. 2 ▸.

First, orientation correction is applied, including image transposition and 90° rotation, to rectify image orientation. Next, intensity normalization is performed: a Gaussian blur with a 9 × 9 kernel is applied using *cv2.GaussianBlur*, followed by z-score normalization (image − μ)/σ, where μ and σ are the image mean and standard deviation, respectively. Pixel values are then linearly scaled to the 0–255 range and converted to uint8 data type.

For noise reduction, fast non-local means denoising is applied using parameters *h* = 10, template window size = 7, and search window size = 21. Subsequently, a Wiener filter is employed with a 9 × 9 Gaussian kernel and *K* = 30 to further suppress residual noise.

Contrast enhancement is achieved using contrast limited adaptive histogram equalization (*CLAHE*) with parameters clipLimit = 2.0 and tileGridSize = (16, 16). Finally, guided filtering is performed using the Wiener-filtered image as the guidance map, with a filter radius of 20 and ɛ = 0.1, to smooth background regions while preserving particle edge details. After preprocessing, the image is resized to 1024 × 1024 pixels as the final input to the Qwen-Image-Edit-2511 model.

### 2.3. Visual large model

**Figure 3:**
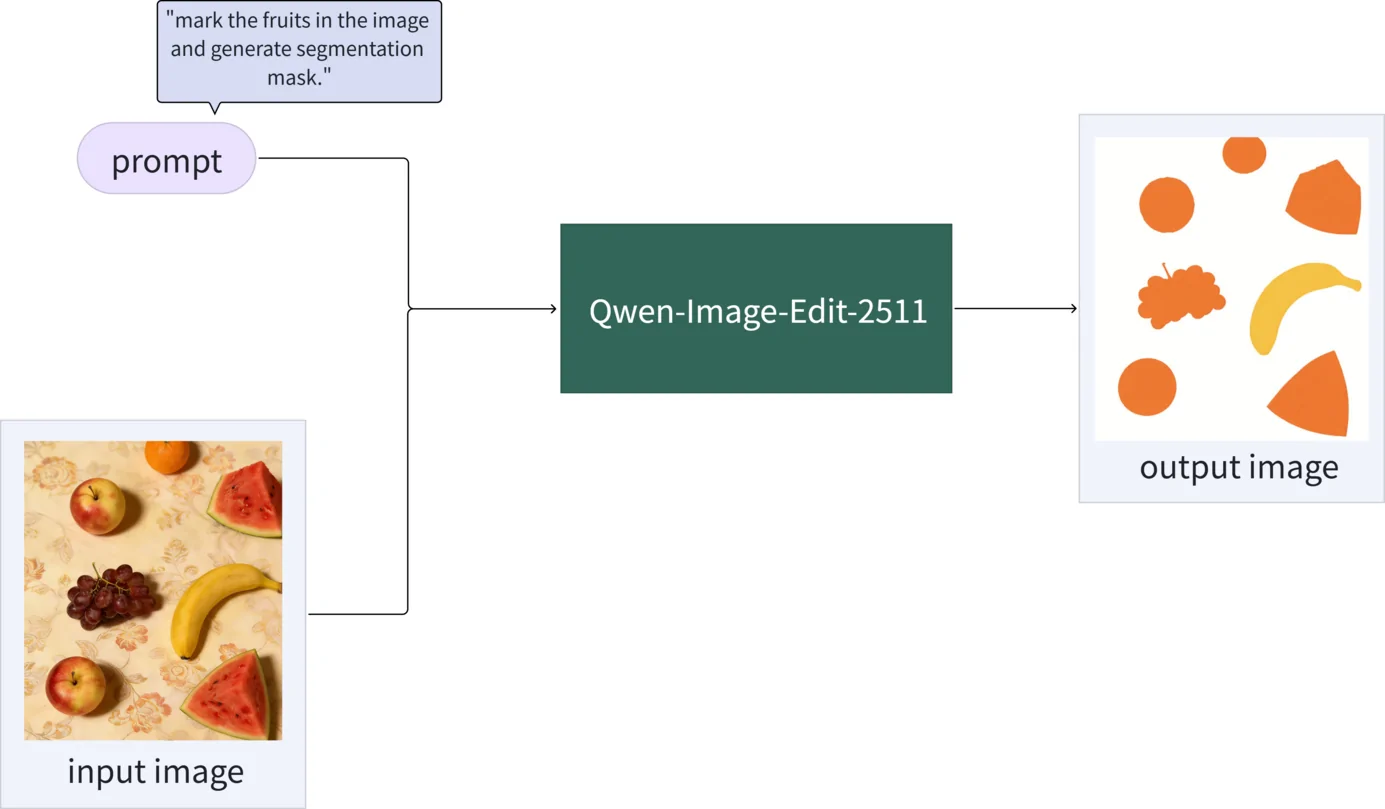
An example of Qwen-Image-Edit-2511 performing image editing based on natural language prompts.

We adopt Qwen-Image-Edit-2511 as the backbone for cryo-EM particle segmentation. Released by the Qwen team in December 2025 ▸, this vision large model is designed for instruction-driven image editing, recognition and segmentation, supporting natural language prompts and demonstrating strong capabilities in fine-grained visual perception. An application example is illustrated in Fig. 3 ▸, showing how the model performs image editing tasks based on natural language prompts.

Its architecture combines instruction-driven control with latent diffusion, integrating several key components: Qwen2.5-VL parses text prompts to generate semantic embeddings; a variational autoencoder (VAE) compresses images into a latent space for denoising and decodes them back to pixel space; a multimodal diffusion transformer (MMDiT) performs latent space denoising with fused text-image representations; and a scheduler balances generation quality with inference efficiency. Additionally, multimodal scalable rotatory positional embedding (MSRoPE) ensures accurate text–image semantic alignment, which is critical for maintaining consistency between the input prompt and the generated output.

Compared with its predecessors, Qwen-Image-Edit-2511 offers improved content consistency and geometric reasoning, reduced image drift, and native support for community low-rank adaptation (LoRA) sub-models, making it well suited for downstream tasks requiring precise structural delineation. This capability is particularly relevant for cryo-EM particle segmentation, where accurate boundary delineation and robustness to structural variability are essential. Among existing visual large models, we selected the Image-Edit variant because its architectural design emphasizes fine-grained semantic understanding and spatial reasoning – characteristics that are hypothesized to be beneficial for distinguishing subtle particle features in noisy cryo-EM micrographs. In natural image tasks, this model demonstrates proficiency in modifying specific regions based on instructions; we hypothesize that this editing capability can be adapted to accurately segment protein particle boundaries, a task that often requires precise pixel-level delineation beyond what traditional bounding-box-based detectors provide.

As a general-purpose model trained primarily on natural images, it has no prior exposure to cryo-EM micrographs. To adapt it to the cryo-EM domain, we perform supervised fine-tuning and reinforcement learning on the CryoPPP benchmark dataset (Section 2.4). This domain-specific fine-tuning aligns the model’s feature extraction capabilities with the unique contrast, noise and structural characteristics of cryo-EM data, enabling it to generate high-fidelity binary masks for protein particles.

### 2.4. Training stage

**Figure 4:**
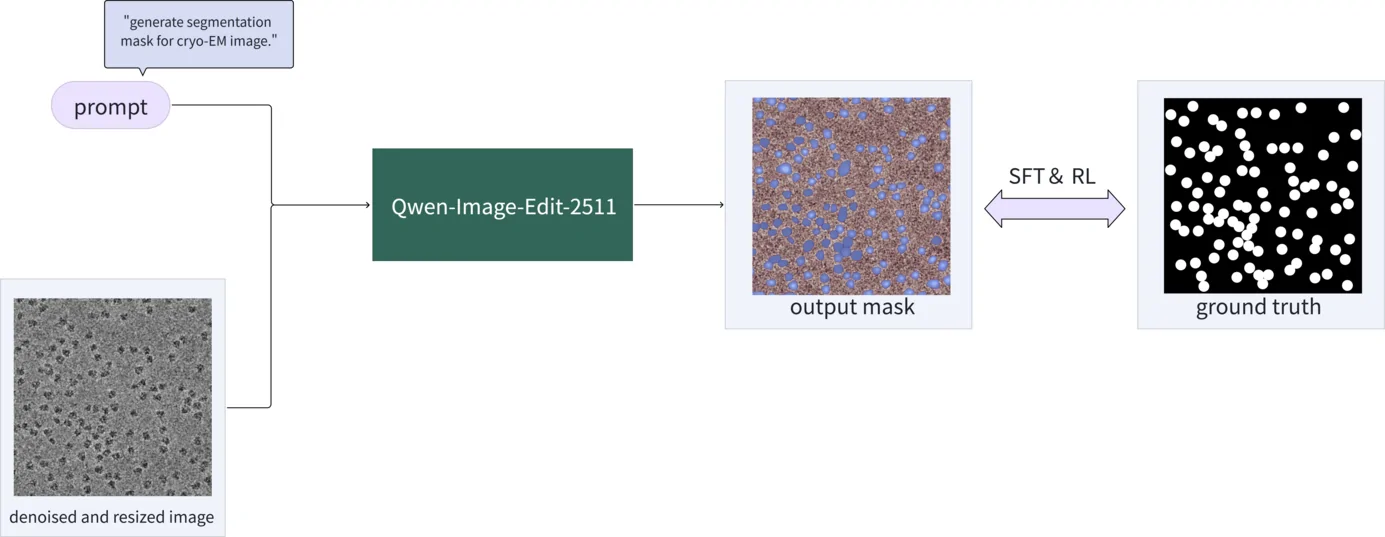
Training stage workflow. The model receives a prompt and image, generates a mask, and iteratively updates parameters via SFT and RL to align the output with the ground truth.

During the training stage, we input the denoised and resized 1024 × 1024 cryo-EM images along with their corresponding masks to the Qwen-Image-Edit-2511 model, and sequentially perform supervised fine-tuning (SFT) and reinforcement learning (RL) to fine-tune the model parameters (the workflow is shown in Fig. 4 ▸). For more details, see Appendix A[App appa].

#### 2.4.1. Supervised fine-tuning (SFT)

Qwen-Image-Edit-2511 is a diffusion model, and conventional supervised fine-tuning (SFT) for such models typically employs denoising loss in the noise space, such as flow matching loss. However, the output of our segmentation task is a binary mask, which differs from the continuous image generation objective the model was originally designed for. To better align the training objective with the task characteristics, we incorporate a Dice loss term in the image space alongside the noise-space loss. The Dice loss is particularly suited for segmentation tasks because it directly measures the overlap between predicted and ground-truth masks, making it inherently robust to class imbalance – a common challenge in cryo-EM particle segmentation where protein particles occupy only a small fraction of the total pixels. Additionally, the Dice loss is more sensitive to the accurate segmentation of small, low-contrast particle regions compared with pixel-wise losses such as cross-entropy.

We fixed the text prompt used during both training and inference to ensure the model executes the segmentation task under a unified instruction. The total SFT loss is composed of two components: the base loss *L*_base_ and the Dice loss *L*_Dice_,
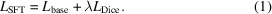
The base loss *L*_base_, calculated in the noise space at each diffusion step, is defined as
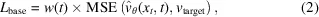
where 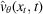 is the model’s predicted flow matching velocity field for the noisy latent representation *x*_*t*_ sampled at timestep *t* and MSE is the mean squared error. For flow matching, we construct a continuous linear interpolation path that transforms the clean latent of ground-truth mask *x*_0_ toward standard Gaussian noise ε, formulated as *x*_*t*_ = (1 − σ_*t*_)*x*_0_ + σ_*t*_ε. Here, σ_*t*_ ∈ [0, 1] is the noise-schedule parameter controlled by timestep *t*: σ_*t*_ = 0 corresponds to the clean ground-truth latent *x*_0_ and σ_*t*_ = 1 corresponds to full Gaussian noise ε.

In flow matching terminology, a velocity field describes the instantaneous rate of change of latent states along the trajectory parameterized by σ_*t*_. The target velocity field *v*_target_ is the analytical derivative of the interpolation trajectory with respect to the noise scale σ_*t*_. Differentiating *x*_*t*_ with respect to σ_*t*_ yields a constant closed-form solution independent of timestep: *v*_target_ = ε − *x*_0_. Crucially, *v*_target_ defines the ideal instantaneous velocity required to move a latent point strictly along our predefined interpolation trajectory. It represents the ground-truth motion vector that would drive the latent variable exactly along the path from clean mask *x*_0_ toward full noise ε. During training, the neural network θ learns to approximate this ideal velocity field by outputting 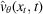 given the noisy latent *x*_*t*_ and timestep *t*. At inference time, integration of the learned velocity field enables the reverse trajectory: starting from pure noise, we follow the learned velocity to recover the clean segmentation mask.

During training, the scheduler directly computes this target vector from *x*_0_, random noise ε and timestep index *t* following standard flow matching rules. The term *w*(*t*) is the scheduler’s training weight (clamped to [0.1, 2.0]) that modulates loss weight by timestep *t* to balance gradient contributions across low-noise and high-noise samples. This weighted MSE loss drives the model to produce velocity predictions that closely match the ideal target motion *v*_target_ at every diffusion step.

To adapt to the binary mask output of the segmentation task, we introduce the Dice loss *L*_Dice_ calculated in the image space as a supplementary loss term. The Dice loss is defined as 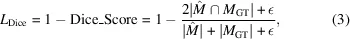
where 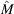 denotes the binary mask predicted by the model at a given timestep; *M*_GT_ represents the ground-truth binary mask; | · | denotes the cardinality (pixel count) of the set; and ε is a small constant (set to 10^−6^ in our experiments) to avoid division by zero. Notably, *L*_Dice_ is computed across all diffusion timesteps rather than only the final step. The weight λ in the total loss is set to 0.5 to balance the contributions of the noise-space MSE loss and the image-space Dice loss.

#### 2.4.2. Reinforcement learning (RL)

Following supervised fine-tuning, we further refine the model using reinforcement learning (RL) to better align its outputs with the practical requirements of particle picking. Unlike SFT, which optimizes the model to match static ground-truth masks, RL allows the model to be guided by reward signals that reflect segmentation quality. This approach is particularly relevant for cryo-EM particle picking, where the ultimate goal is to select particles that contribute to high-quality 3D reconstructions, a criterion that may not be fully captured by pixel-wise mask accuracy alone. By framing particle picking as a sequential decision-making process, RL enables the model to learn strategies that prioritize segmentation outputs with favorable characteristics, such as well defined boundaries and minimal fragmentation, through iterative optimization guided by a designed reward function.

We design a composite loss function for RL fine-tuning, with the total loss defined as
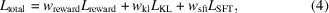
where the default weights are set as *w*_reward_ = 1.0, *w*_kl_ = 0.01, and *w*_sft_ = 0.1. Each loss component serves a distinct purpose:

*Reward loss* (*L*_reward_): aimed at maximizing the segmentation quality. We define: 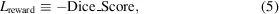
where Dice_Score measures the overlap between the predicted mask and the ground truth. Minimizing this loss encourages the model to produce masks with higher Dice similarity.

*KL loss* (*L*_KL_): serves as a regularization term to prevent catastrophic forgetting of knowledge learned during SFT, ensuring training stability. It is calculated as the mean squared error (MSE) between the model’s noise prediction and the training target from a reference model: 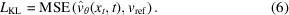 If no reference model is provided, the SFT-fine-tuned model serves as the reference.

*SFT loss* (*L*_SFT_): retained with a weight of 0.1 to preserve the basic noise prediction ability essential for the diffusion process of Qwen-Image-Edit-2511, as defined in equation (1).

This RL fine-tuning strategy complements the objectives of supervised fine-tuning by introducing a reward-driven optimization pathway. While SFT establishes a foundation of mask prediction accuracy, RL encourages the model to further refine its outputs toward segmentation characteristics that are desirable for downstream particle picking. The combination of these two stages enables the model to generate high-fidelity binary masks that are both accurate and well suited for subsequent coordinate extraction.

### 2.5. Inference stage

**Figure 5:**
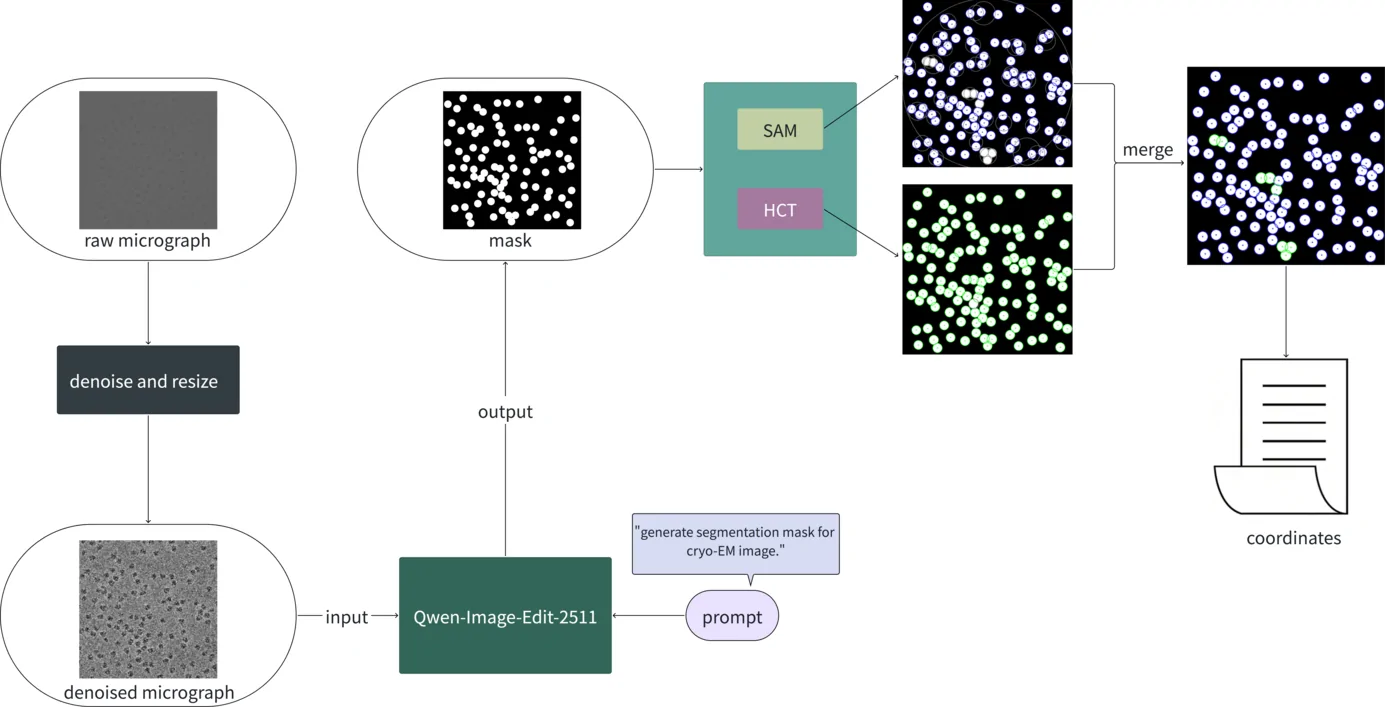
Inference stage workflow. The fine-tuned model generates a binary mask, which is simultaneously processed by SAM and HCT; their outputs are fused to obtain final particle coordinates.

The inference workflow is illustrated in Fig. 5 ▸. During inference, the denoised cryo-EM micrographs, resized to 1024 × 1024 pixels, are input to the fine-tuned Qwen-Image-Edit-2511 model together with a fixed text prompt (consistent with the training stage). The model directly generates a binary mask corresponding to protein particle regions.

To derive precise particle coordinates from the generated mask, we employ a dual-branch post-processing pipeline that combines two complementary modules: the segment anything model (SAM) and the Hough circle transform (HCT). SAM is a general-purpose image segmentation model designed for prompt-driven segmentation tasks. Its architecture comprises three key components: an image encoder that extracts high-dimensional visual embeddings from the input; a prompt encoder that encodes user-provided prompts (such as points or bounding boxes) into semantic embeddings; and a mask decoder that fuses these representations to generate segmentation masks. In this workflow, SAM processes the binary mask to produce fine-grained boundary segmentations of particle regions. SAM is particularly effective at delineating detailed particle edges when provided with appropriate spatial prompts. HCT is a classic geometric feature detection algorithm that identifies circular or near-circular structures. It operates by mapping edge pixels from the image space to a parameter space of center coordinates (*x*, *y*) and radius *r*, where local maxima in this parameter space correspond to detected circles. This method relies on geometric characteristics rather than semantic context, making it especially suitable for identifying circular protein particles, including those in overlapping configurations where boundaries may be ambiguous.

The motivation for this dual-branch design lies in the distinct and complementary characteristics of the two approaches: SAM provides fine-grained boundary delineation, while HCT offers geometric detection of circular structures. This combination is intended to translate the semantic-rich masks from the visual large model into coordinates that meet the geometric precision required for downstream 3D reconstruction.

The mask is simultaneously processed by both modules, and their outputs are fused to generate the final particle coordinates. In this fusion strategy, HCT-derived centroids serve as spatial anchors to guide SAM’s boundary segmentation, while SAM’s refined boundaries help validate HCT detections. The fusion process eliminates redundant coordinates and corrects positional discrepancies, yielding a refined set of accurate particle center coordinates. This complementary design aims to achieve robust localization across a range of cryo-EM micrograph conditions, including crowded fields where particle overlap is common, by leveraging the strengths of both semantic boundary delineation and geometric feature detection.

### 2.6. Evaluation metrics for particle picking

Following particle picking, the coordinates of selected protein particles were compared against the ground-truth annotations provided by the CryoPPP dataset. A picking result was considered correct if the Euclidean distance between the predicted particle coordinate and the ground-truth coordinate was less than one-quarter of the box size. Three standard metrics were employed to quantify particle picking performance: precision, recall and *F*_1_ score.

Precision is defined as the ratio of correctly identified true particles (true positives, TP) to the total number of picked particles (TP + false positives, FP), reflecting the accuracy of the picking results: 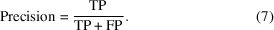
Recall is defined as the ratio of correctly identified true particles (TP) to the total number of actual true particles (TP + false negatives, FN), indicating the model’s ability to capture true particles: 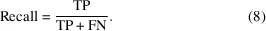
The *F*_1_ score, defined as the harmonic mean of precision and recall, provides a balanced assessment of overall particle picking performance: 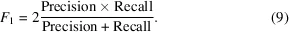
In addition to these metrics, the impact of particle picking on downstream structural determination was assessed through 3D reconstruction. For each method, the selected particles were subjected to *ab initio* reconstruction, homogeneous refinement and non-uniform refinement in *CryoSPARC*. The global resolution of the resulting density maps, reported according to the gold-standard Fourier shell correlation (FSC) criterion at 0.143, served as an additional evaluation metric that directly reflects the structural biology outcomes of particle selection.

## 3. Results

### 3.1. Computational overhead and runtime

We summarize the computational hardware and runtime consumption of *CryoQSeg* during training and inference in Table 1 ▸. Multi-GPU parallelism can be adopted to accelerate all stages above for higher throughput.

Specifically, generating segmentation masks via the large vision model can be accelerated by multi-GPU parallel computation. The coordinate extraction step based on SAM and Hough circle transform is also parallelizable across multiple GPUs to improve overall processing speed.

### 3.2. Particle picking performance

We evaluated the particle picking performance of *CryoQSeg* on five protein datasets derived from the CryoPPP test dataset. Table 2 ▸ summarizes the characteristics of these datasets, including protein type, number of labeled images and number of labeled particles. Performance was quantified using precision, recall and *F*_1_ score, following the evaluation protocol described in Section 2.6.

For comparison, we selected four representative baseline methods: blob detection, template matching, *Topaz* and *CryoSegNet*. Blob detection and template matching are built-in particle picking algorithms in widely used cryo-EM software packages such as *CryoSPARC* and *RELION*, representing conventional approaches commonly employed in practice. *Topaz*, also integrated into these platforms, offers a deep-learning-based alternative with positive-unlabeled learning. *CryoSegNet* is a recent deep-learning-based method that combines an attention-gated U-Net with the segment anything model (SAM), and has been shown to outperform several existing approaches in its original study. Together, these four methods encompass both routine practical tools and recent advances in the field, providing a well grounded basis for evaluating *CryoQSeg*.

Table 3 ▸ presents a quantitative comparison of *CryoQSeg* against these baseline methods across the five test datasets, reporting precision, recall and *F*_1_ score for each method.

Across the five datasets, *CryoQSeg* achieved competitive performance relative to the baseline methods. On datasets EMPIAR 10028, 10081 and 11056, *CryoQSeg* obtained the highest *F*_1_ scores among all methods tested. Notably, on EMPIAR 10028, *CryoQSeg* achieved a recall of 0.977, indicating strong capability in recovering true particle signals. On EMPIAR 10017, *CryoQSeg* attained a precision of 0.769, reflecting its ability to maintain low false-positive rates.

The performance characteristics of the different methods varied across datasets. For instance, blob detection and template matching tended to achieve high recall but lower precision on several datasets, indicating a trade-off toward capturing many candidates at the cost of increased false positives. *CryoSegNet*, in contrast, showed higher precision but lower recall on certain datasets, such as EMPIAR 10532, reflecting a different balance between sensitivity and specificity. *CryoQSeg* exhibited a more balanced profile across datasets, with both precision and recall contributing to its *F*_1_ scores.

**Figure 6:**
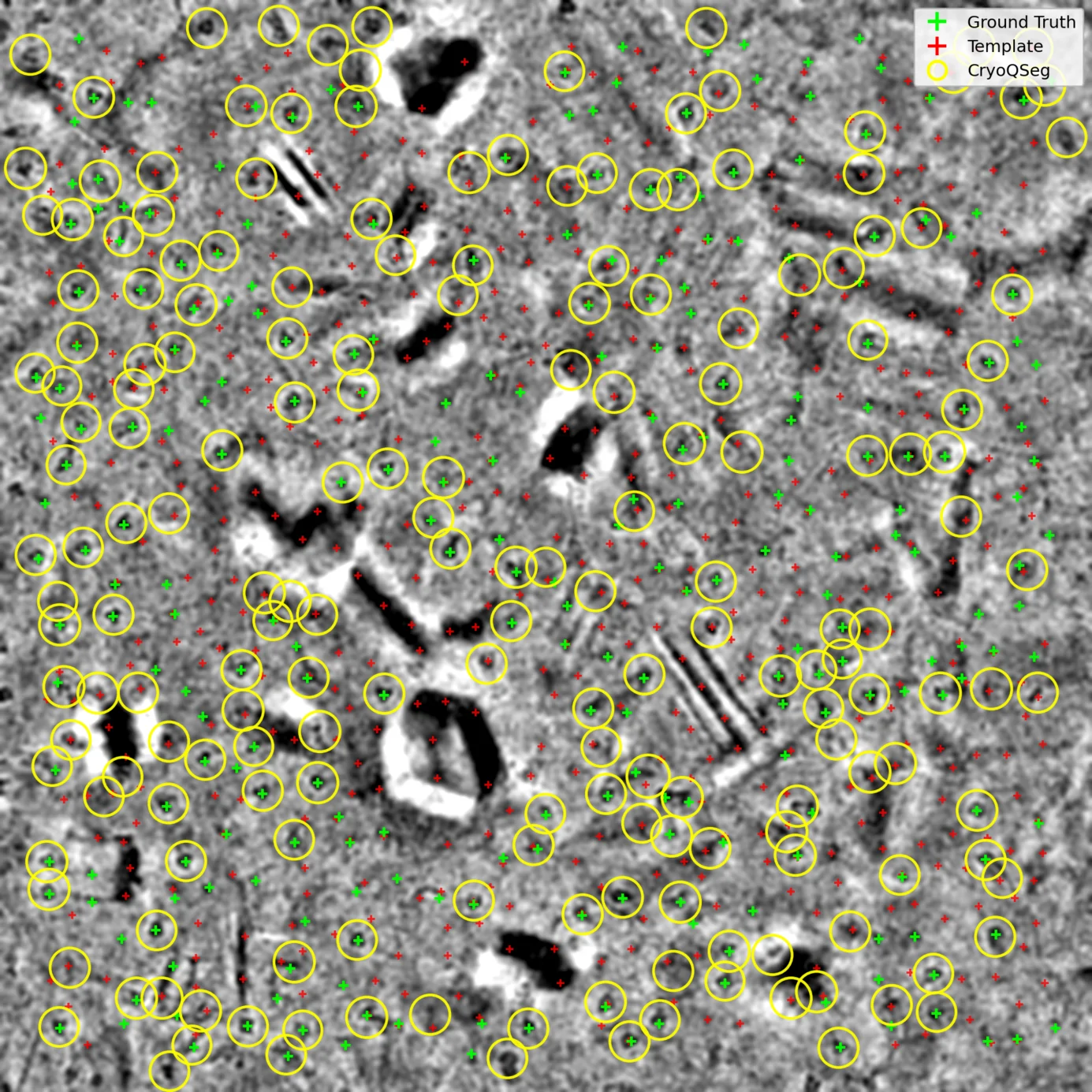
Visual particle picking comparison on a representative micrograph from the low-signal EMPIAR 10532 dataset. Green plus signs denote ground-truth particle positions; red plus signs represent picking outputs of the template baseline; yellow circles indicate particles detected by *CryoQSeg*. Numerous faint, low-contrast true particles are successfully identified by *CryoQSeg* yet missed by template picking. Moreover, the template method frequently misclassifies contamination and ice-artifact regions as valid particles, while *CryoQSeg* largely avoids such false-positive predictions.

On the challenging low-signal dataset EMPIAR 10532, *CryoQSeg* achieved an *F*_1_ score of 0.389, compared with 0.208 for *CryoSegNet* and 0.150 for *Topaz*, suggesting robustness in scenarios where particle signals are weak. To provide intuitive visual evidence for this performance difference, we select one representative micrograph from EMPIAR 10532 for qualitative comparison, as shown in Fig. 6 ▸. We compare ground-truth annotations, outputs from the template method (which yields the best performance among baselines on this dataset), together with detection results predicted by *CryoQSeg*.

Similarly, on EMPIAR 11056, which features complex background characteristics, *CryoQSeg* obtained an *F*_1_ score of 0.493, the highest among the compared methods.

These results indicate that *CryoQSeg*, by leveraging a visual large model backbone tailored to cryo-EM data, offers a viable approach to particle picking that balances sensitivity and specificity across diverse datasets, establishing a quantitative foundation for its practical utility.

In addition, we examined the impact of particle selection on 3D reconstruction resolution. However, given the substantial disparity in particle counts across methods (*e.g.* 45 218 versus 26 356 on EMPIAR 10028), resolution differences cannot be unambiguously attributed to picking quality alone. This analysis is therefore presented as supplementary material in Appendix B[App appb] and C[App appc], serving as an exploratory observation rather than a controlled comparison.

Furthermore, we supplemented extra benchmark experiments to compare *CryoQSeg* with two recently proposed segmentation-based particle picking algorithms, *UPicker* (Zhang *et al.*, 2025 ▸) and *VMPicker* (Zhu *et al.*, 2026 ▸), to enrich the validity of our performance evaluation. Full experimental details, training configurations and quantitative comparison results on all test datasets are available in Appendix D[App appd].

### 3.3. Application to real-world cryo-EM data

To evaluate the practical utility of *CryoQSeg*, we applied it to the Crt-SPARTA-gRNA-tDNA protein dataset (Gao *et al.*, 2024 ▸). This dataset was not used during fine-tuning and it was selected for three considerations. First, it contains three distinct oligomeric conformations – monomer, dimer and tetramer – coexisting within the same sample, enabling assessment of whether a single model, without parameter adjustment, can consistently pick particles of varying sizes and shapes. Second, the original study employed multiple particle picking strategies (including template matching, *Topaz* and *crYOLO*) to achieve high-resolution reconstructions, rendering this dataset a representative benchmark for comparing picking outcomes across diverse methodological approaches. Third, unlike the CryoPPP datasets used in Section 3.1, this dataset lacks publicly available ground-truth particle coordinates, precluding quantitative evaluation via precision, recall or *F*_1_ score. Consequently, we adopted the resolution of the final 3D density maps as the primary metric for this evaluation. While resolution is influenced by multiple factors beyond particle selection – including particle orientation distribution and data quantity – it provides a practical measure of the overall utility of the picked particles for downstream structural determination, particularly when comparing reconstructions derived from the same model under different input conditions.

Our experiment was conducted using a subset of 2652 micrographs, fewer than the 7330 to 20 299 micrographs used in the original study. Using particles picked by *CryoQSeg*, we performed 3D *ab initio* reconstruction and homogeneous refinement in *CryoSPARC*. Table 4 ▸ summarizes the reconstruction results, including the number of micrographs used, the number of particles selected and the achieved resolutions for each oligomeric state, alongside the corresponding values reported in the original study for reference.

The resulting density maps yielded resolutions of 3.39 Å for the monomer, 4.43 Å for the dimer and 4.30 Å for the tetramer. For reference, the original study reported resolutions of 3.27 Å (monomer), 3.49 Å (dimer) and 3.41 Å (tetramer) using larger micrograph sets.

**Figure 7:**
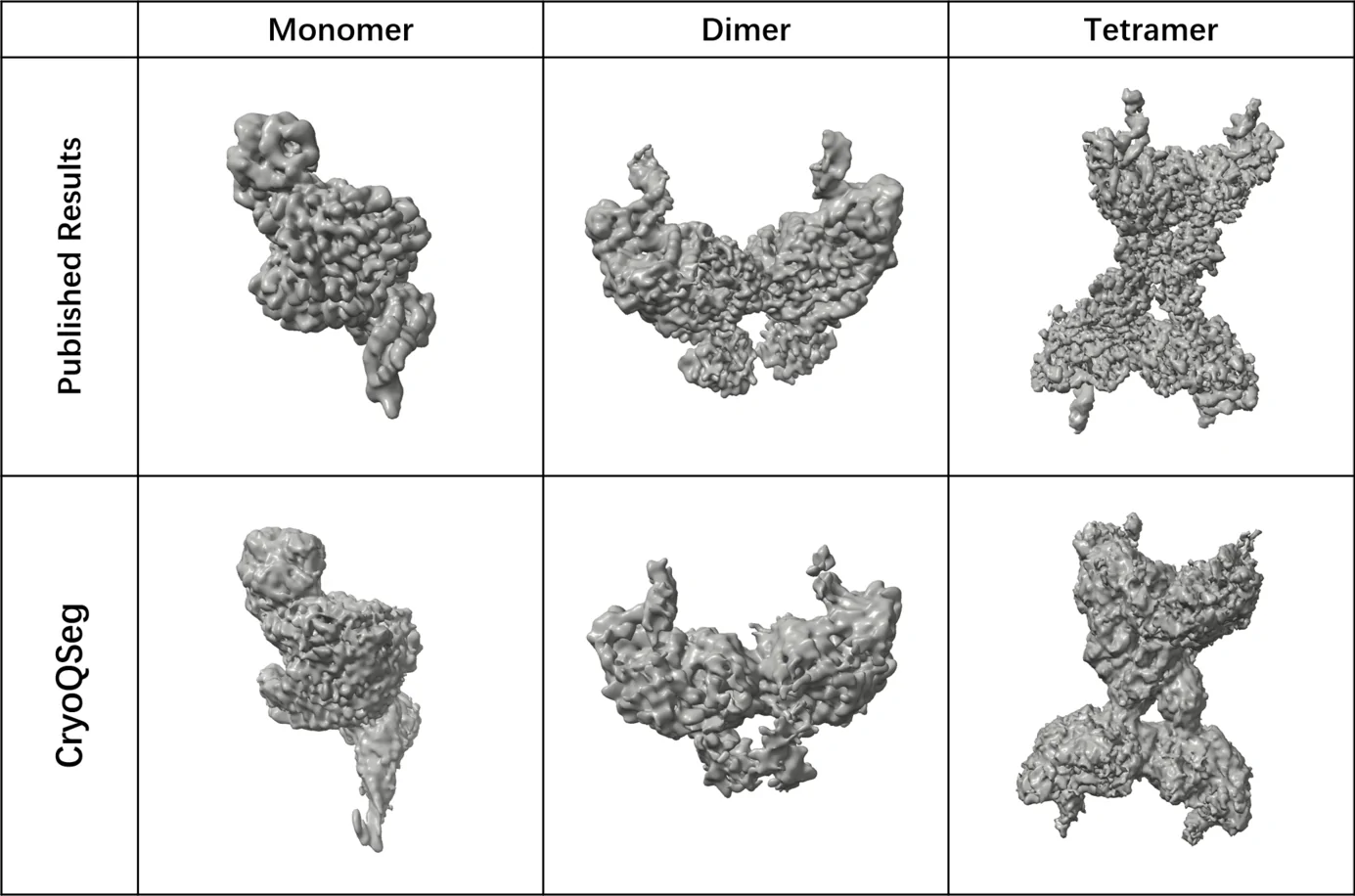
Density map comparison of Crt-SPARTA-gRNA-tDNA oligomers.

Fig. 7 ▸ presents the reconstructed density maps. Despite the use of fewer micrographs, the maps generated from *CryoQSeg*-picked particles (lower row) exhibit well defined structural features, including domain boundaries in the monomer and oligomeric interfaces in the dimer and tetramer, with detail comparable to the published maps (upper row).

**Figure 8:**
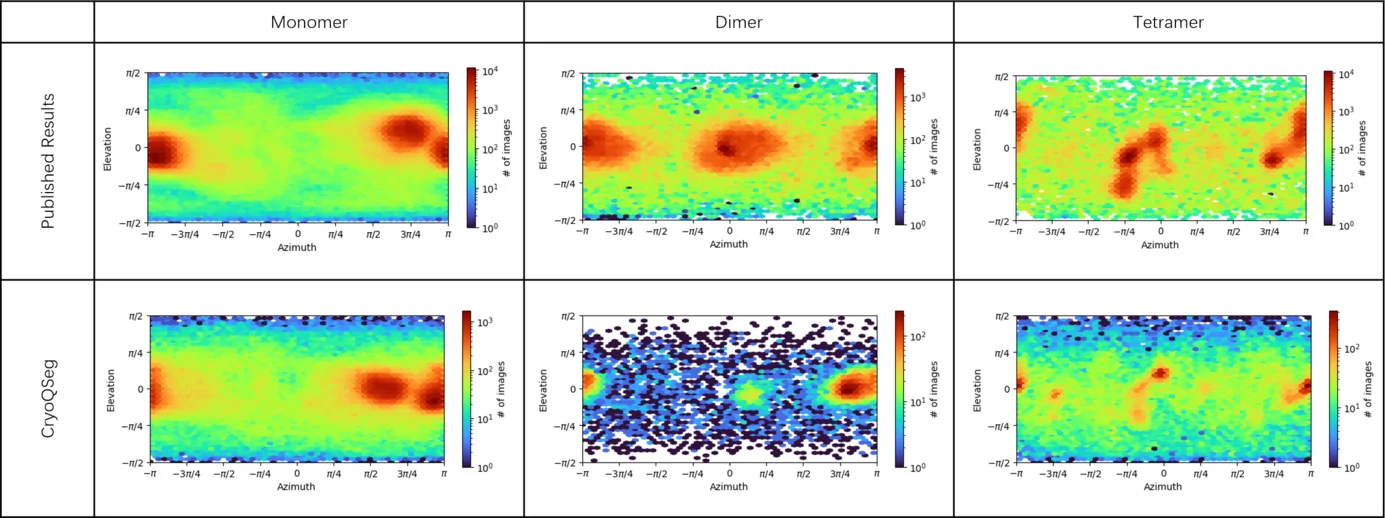
Particle orientation-distribution heat maps comparing published reference results and particles picked by *CryoQSeg* for monomer, dimer and tetramer assemblies. Horizontal axis: azimuth angle; vertical axis: elevation angle. The color intensity corresponds to particle count within each angular bin on a logarithmic scale.

To assess whether the subtle preferred-orientation features observed in our density maps (*e.g.* the top part of the monomer and the bottom of the dimer) arise from the picking model or reflect intrinsic specimen properties, we compared the Euler-angle distributions of particles selected by *CryoQSeg* with those reported in the original study (Fig. 8 ▸). The angular coverage patterns are highly consistent between the two sets: both exhibit similar densely and sparsely sampled regions, indicating that the orientation distribution is inherent to the cryo-EM specimen rather than artificially introduced by *CryoQSeg*. Notably, the original work achieved high-resolution reconstructions using 0.6–1.1 million particles, whereas our *CryoQSeg*-based reconstruction used only 12 000–223 000 particles per oligomeric state – roughly one order of magnitude fewer. Despite this severe data limitation, we obtained resolutions of 3.39 Å (monomer), 4.43 Å (dimer) and 4.30 Å (tetramer). The fact that *CryoQSeg* can produce reasonably high-resolution maps under such particle-starved conditions, while faithfully recovering the specimen’s intrinsic orientation distribution, suggests that our method does not introduce severe picking bias. The minor orientation-related artifacts we observe are most likely attributable to limited particle statistics rather than a systematic failure of the picking algorithm.

These observations suggest that *CryoQSeg* is capable of handling multi-conformation datasets and producing particle selections that support high-quality 3D reconstruction, even with limited input data. Together with the quantitative results in Section 3.1, this case study demonstrates the potential of *CryoQSeg* as a practical and adaptable tool for cryo-EM particle picking across diverse experimental conditions.

### 3.4. HCT-assisted SAM fusion strategy

To evaluate the contribution of the HCT-assisted SAM fusion strategy to overall particle picking performance, we conducted a comparative experiment between the full dual-branch pipeline (HCT-assisted SAM) and a variant using SAM alone on the five test datasets. The quantitative results are presented in Table 5 ▸.

Across all datasets, the HCT-assisted SAM method achieved higher recall and *F*_1_ scores compared with the SAM-only variant. On dataset EMPIAR 10017, the SAM-only method achieved a precision of 0.756, recall of 0.409 and an *F*_1_ score of 0.531, while the HCT-assisted SAM improved recall to 0.584 and the *F*_1_ score to 0.664. On dataset EMPIAR 10028, both methods maintained high recall (0.974 for SAM-only versus 0.977 for HCT-assisted SAM), with the latter achieving a slightly higher precision (0.739 versus 0.728) and *F*_1_ score (0.841 versus 0.834). On dataset EMPIAR 10081, HCT-assisted SAM improved recall from 0.834 to 0.889 and the *F*_1_ score from 0.714 to 0.741.

On the challenging low-signal dataset EMPIAR 10532, the SAM-only method achieved a marginally higher precision (0.457 versus 0.454), but HCT-assisted SAM improved recall from 0.278 to 0.341 and the *F*_1_ score from 0.346 to 0.389. On dataset EMPIAR 11056, which features complex background characteristics, HCT-assisted SAM increased recall from 0.257 to 0.420, precision from 0.562 to 0.598 and the *F*_1_ score from 0.353 to 0.493.

These results indicate that the dual-branch fusion strategy, which combines SAM’s boundary delineation capability with HCT’s geometric detection of circular structures, contributes to improved particle recovery, particularly in scenarios involving overlapping particles or low-signal conditions. The complementary design enables a more balanced trade-off between precision and recall across diverse cryo-EM datasets.

### 3.5. Effect of reinforcement learning fine-tuning

To evaluate the contribution of reinforcement learning (RL) fine-tuning to overall particle picking performance, we conducted a comparative experiment between the full two-stage training pipeline (SFT+RL) and a baseline model with only supervised fine-tuning (SFT alone) on the five test datasets. The quantitative results are presented in Table 6 ▸.

Across all datasets, the SFT+RL framework consistently achieves higher recall and *F*_1_ scores than the SFT-only baseline while maintaining comparable precision. On dataset EMPIAR 10017, the two methods obtain identical precision (0.769), while SFT+RL improves recall from 0.554 to 0.584 and the *F*_1_ score from 0.644 to 0.664, demonstrating enhanced particle recovery without compromising precision. On dataset EMPIAR 10028, SFT+RL increases recall from 0.938 to 0.977 and the *F*_1_ score from 0.832 to 0.841, exhibiting stronger capability in capturing large-sized protein particles.

On dataset EMPIAR 10081, SFT+RL elevates recall from 0.828 to 0.889 and the *F*_1_ score from 0.728 to 0.741, with a minor reduction in precision (from 0.650 to 0.635). For the challenging low-signal dataset EMPIAR 10532, SFT+RL maintains the same precision (0.454) as SFT alone, while improving recall from 0.292 to 0.341 and the *F*_1_ score from 0.355 to 0.389 – an important improvement for detecting faint particles. On dataset EMPIAR 11056 with complex backgrounds, SFT+RL achieves higher recall and *F*_1_ score compared with SFT alone, with a negligible reduction in precision (from 0.603 to 0.598), further validating its robustness.

These results demonstrate that RL fine-tuning effectively optimizes the model for practical particle picking tasks. By integrating reward-guided optimization, the SFT+RL pipeline achieves a more balanced trade-off between precision and recall, outperforming the SFT-only method across all test datasets – particularly in scenarios involving low-signal particles, where the improvement in recall is most pronounced.

### 3.6. Ice contamination avoidance

Beyond quantitative metrics, a key practical advantage of *CryoQSeg* is its robustness to ice contamination – a common challenge in cryo-EM that often leads to false positives in particle picking. Benefiting from the powerful semantic representation of the visual large model, *CryoQSeg* effectively distinguishes genuine protein particles from ice artifacts without requiring explicit negative samples during training.

**Figure 10:**
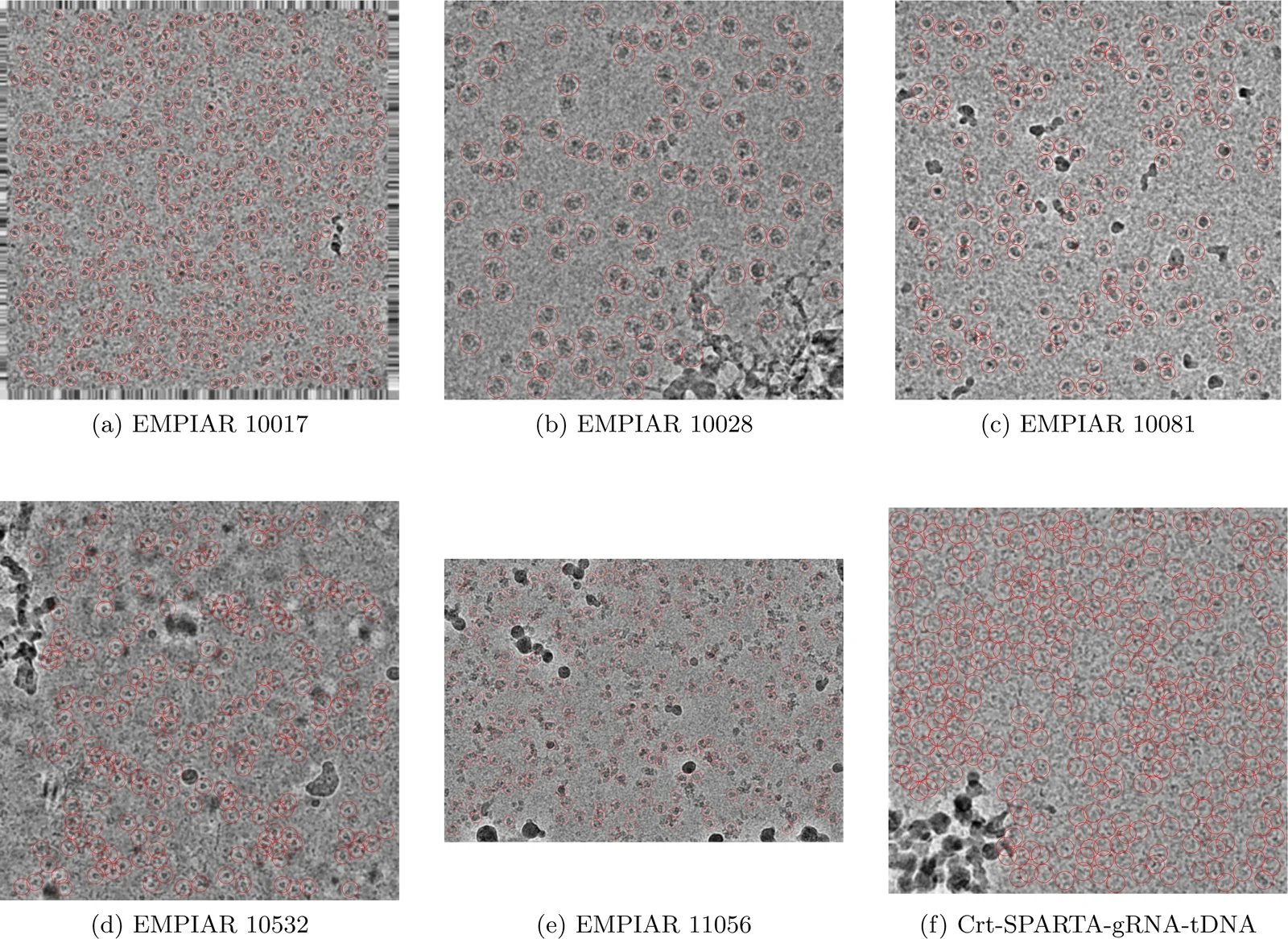
Qualitative particle picking results of *CryoQSeg* on six datasets with ice contamination. Red circles represent the protein particles detected by *CryoQSeg*, which are accurately localized in valid regions and avoid ice artifacts. (*a*)–(*e*) correspond to five CryoPPP benchmark datasets and (*f*) denotes the Crt-SPARTA-gRNA-tDNA dataset.

Fig. 10 in Appendix E[App appe] presents qualitative particle picking results across all six benchmark datasets, including five CryoPPP datasets and the Crt-SPARTA-gRNA-tDNA complex. In micrographs with severe ice contamination, *CryoQSeg* consistently avoids ice-covered regions and accurately localizes true particles, demonstrating strong generalization to challenging imaging conditions. These observations complement the quantitative results in Sections 3.1–3.4 and underscore the practical utility of *CryoQSeg* for routine cryo-EM workflows.

## 4. Discussion

*CryoQSeg* advances cryo-EM particle picking via a synergistic technical framework, whose performance across challenging datasets (*e.g.* the multi-conformation Crt-SPARTA-gRNA-tDNA) stems from four interconnected, mechanistically grounded innovations that address the field’s core trade-offs between sensitivity, specificity and practicality.

### 4.1. Practical utility and compatibility

*CryoQSeg* is designed with practical adoption in mind. It requires no additional labeled data beyond the public CryoPPP dataset used for fine-tuning, leveraging the pre-trained visual large model’s general feature extraction capabilities. The output particle coordinates are formatted as star files, which are compatible with mainstream cryo-EM reconstruction platforms such as *CryoSPARC* and *RELION*. This compatibility facilitates seamless integration into existing cryo-EM workflows, allowing direct progression from particle picking to 3D reconstruction.

A notable feature of *CryoQSeg* is that the same model configuration – with fixed hyperparameters and without per-dataset tuning – was applied uniformly across all benchmark datasets in our evaluation (Section 3.1) and the Crt-SPARTA-gRNA-tDNA dataset in Section 3.2. This consistency demonstrates the model’s ability to generalize to unseen samples and imaging conditions without requiring dataset-specific adjustments. This contrasts with many existing methods that often require dataset-specific parameter adjustments or retraining to achieve optimal performance. The ability to maintain consistent performance across diverse protein types, imaging conditions and particle sizes – exemplified by the successful application to the Crt-SPARTA-gRNA-tDNA complex (Section 3.2), which contains monomeric, dimeric and tetrameric particles of varying sizes – underscores the generalization capability of the visual large model backbone. This capability is particularly relevant for samples exhibiting conformational heterogeneity, a common challenge in structural biology that often requires manual intervention or dataset-specific tuning with conventional approaches.

The practical utility of this design is further illustrated by the reconstruction results on the Crt-SPARTA-gRNA-tDNA complex, where *CryoQSeg* produced particle selections that supported high-resolution 3D reconstructions (3.39 Å for the monomer, 4.43 Å for the dimer and 4.30 Å for the tetramer) using only 2652 micrographs – substantially fewer than the 7330–20 299 micrographs employed in the original study. This suggests that *CryoQSeg* can deliver reliable particle selections even with limited input data, enhancing its practicality for routine structural biology applications.

### 4.2. Limitations

Despite its demonstrated performance, *CryoQSeg* has several limitations that warrant consideration. First, the framework is computationally intensive. Specifically, generating a segmentation mask from a single preprocessed cryo-EM micrograph using the large vision model on a single NVIDIA A100 GPU takes approximately 90 s. This inference procedure can be accelerated through parallel computation across multiple GPUs. After obtaining the segmentation mask, the post-processing step that extracts particle coordinates via SAM and Hough circle transform (HCT) requires around 10 s per micrograph, which can also be parallelized across multiple GPUs to improve throughput. Even with such optimizations, the overall sequential pipeline remains substantially slower than traditional particle picking tools. This high computational cost stems from the large model backbone and the sequential post-processing pipeline, limiting scalability for datasets comprising tens of thousands of micrographs. It should be noted, however, that the current implementation is a proof-of-concept prototype; no systematic optimization for inference speed has been applied. The primary contribution of this work lies in demonstrating the feasibility of adapting visual large models to cryo-EM particle picking, and the reported runtime reflects the performance of an unoptimized prototype rather than the theoretical upper bound of the approach.

Second, the feature decision-making process within the visual large model remains largely opaque. The specific semantic features that drive particle detection are not readily interpretable, making it difficult to diagnose systematic failure modes – for instance, consistent mispicking of a particular particle type or systematic omission under specific imaging conditions.

Third, while *CryoQSeg* exhibits strong generalization across the datasets evaluated, its performance on extremely rare particle morphologies or non-globular shapes has not been systematically tested. The current evaluation is limited to the protein types represented in the CryoPPP benchmark and the Crt-SPARTA-gRNA-tDNA complex.

Finally, while the visual large model demonstrates strong generalization, its performance is influenced by the choice of natural language prompts, as discussed in Appendix F[App appf], suggesting that prompt design remains an open aspect for further investigation.

### 4.3. Future work

Building on the current results, several directions for future research are evident. A primary priority is reducing computational cost. We plan to explore model compression techniques, including knowledge distillation to train a lightweight student model that approximates the performance of the current large model, and model quantization to reduce memory footprint and accelerate inference by converting 32-bit floating-point weights to 8-bit integers. Parallelization of the post-processing pipeline, overlapping HCT and SAM inference steps, is also under consideration. The target is to reduce per-micrograph processing time to under 10 seconds on an A100 GPU while retaining at least 95% of the current model’s performance.

Enhancing model interpretability represents another important direction. Feature attribution analysis could be employed to map latent features to structural particle properties, offering insights into the model’s decision-making process and aiding in the diagnosis of failure cases.

Our exploratory analysis in Appendix F[App appf] reveals that the performance of the visual large model is sensitive to the design of natural language prompts, with the optimal prompt varying across datasets. While the current framework uses a fixed instruction to ensure consistency, systematically investigating prompt engineering – such as adaptive prompt selection or learnable prompts – may further enhance generalization and reduce dataset-specific tuning. We consider this an important direction for future work toward more robust vision-language model-based particle picking.

Beyond algorithm-oriented optimizations, we are developing a graphical user interface (GUI) to enable more intuitive operation for end users. We also intend to publicly release the model and full software package to the research community in the near future.

Finally, extending the framework to three-dimensional cryo-electron tomography (cryo-ET) volumes would broaden its utility for *in situ* structural biology, enabling particle picking directly from tomographic reconstructions. Addressing these directions would further establish *CryoQSeg* as a versatile and reliable tool for structural biology research.

## 5. Conclusion

In this work, we explored the application of a visual large model to cryo-EM particle picking, presenting *CryoQSeg*as a framework that integrates Qwen-Image-Edit-2511 with a hybrid post-processing pipeline combining Hough circle detection and the segment anything model. By adapting a general-purpose vision model to the cryo-EM domain through supervised fine-tuning with a combined noise-space and image-space loss, followed by reinforcement-learning-based refinement, we demonstrate that such models can be tailored to generate high-fidelity particle masks from raw micrographs.

Quantitative evaluation on the CryoPPP benchmark dataset shows that *CryoQSeg* achieves competitive performance relative to established particle picking methods, including blob detection, template matching, *Topaz* and *CryoSegNet*. Application to the Crt-SPARTA-gRNA-tDNA dataset further illustrates the framework’s potential to handle multi-conformation samples and support high-resolution 3D reconstruction with limited input data. Beyond these quantitative gains, *CryoQSeg* effectively avoids ice-contaminated regions during particle picking.

Several limitations remain, including computational efficiency and model interpretability, which point toward future research directions such as model compression, knowledge distillation and extension to cryo-electron tomography. Overall, this study presents a methodological exploration of how modern visual large models can be adapted for cryo-EM particle picking, laying a foundation for further development of foundation-model-based cryo-EM image-processing approaches in structural biology.

## Supplementary Material

Dataset for the study: https://github.com/BioinfoMachineLearning/cryoppp

## Figures and Tables

**Figure 9:**
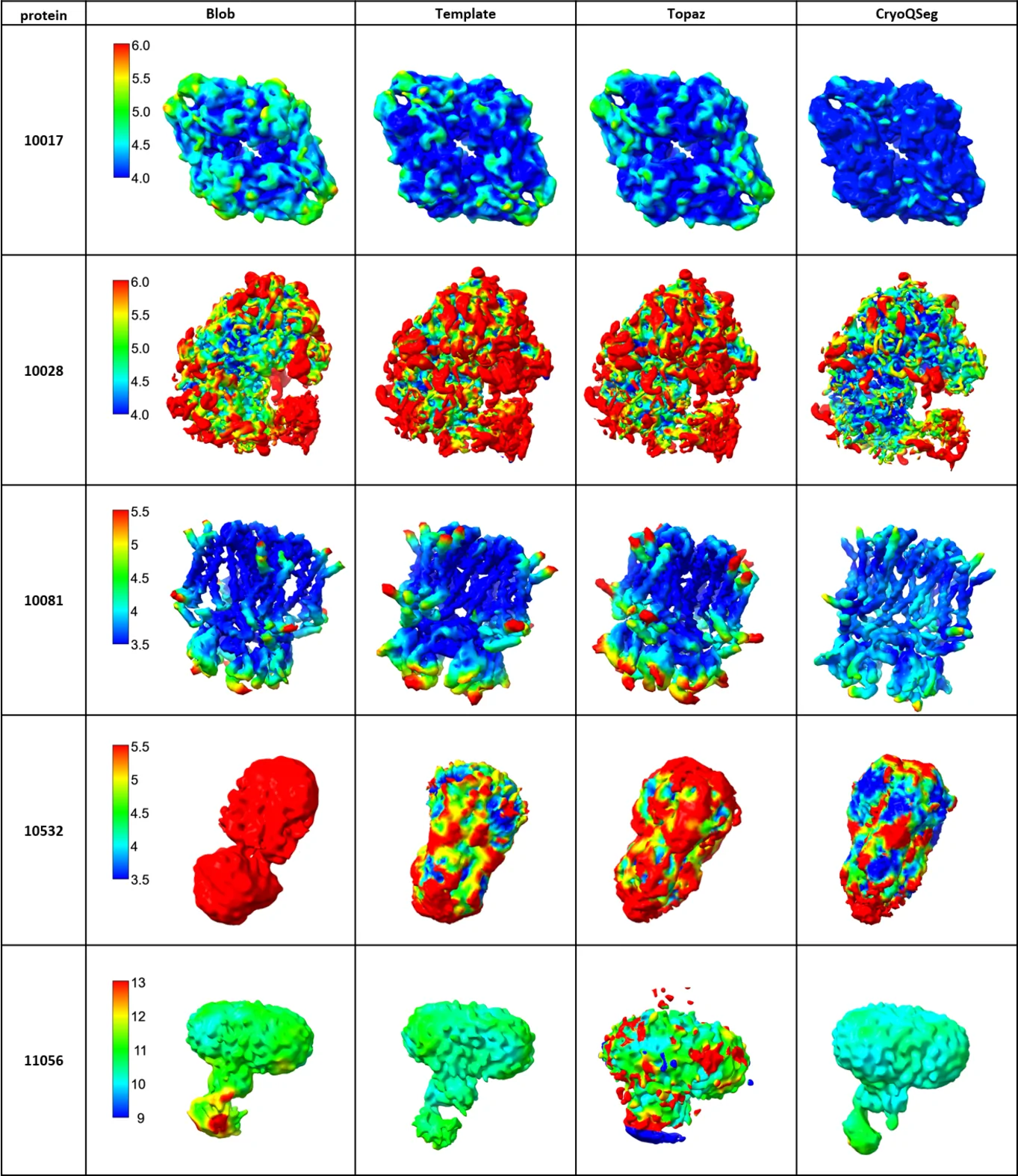
Local-resolution-colored 3D density maps for reconstructions obtained using blob detection, template matching, *Topaz* and *CryoQSeg* across five EMPIAR datasets. Each density map is colored on a per-voxel basis according to its local resolution. The color bars indicate local-resolution values in ångströms, where smaller values (blue color) represent better-resolved map regions. Note that reconstructed density maps of *CryoSegNet* were not publicly available and are therefore excluded in this local-resolution comparison.

**Table 1:** Computational resource and runtime consumption of *CryoQSeg*

Stage	Hardware configuration	Runtime
SFT training	6 × NVIDIA A100 (80 GB) GPU	∼20 hours
RL fine-tuning	6 × NVIDIA A100 (80 GB) GPU	∼8 hours
Mask generation (inference)	Single NVIDIA A100 (80 GB) GPU	∼90 s per micrograph
SAM+HCT post-processing	Single NVIDIA A100 (80 GB) GPU	∼10 s per micrograph

**Table 2:** Test datasets Datasets are identified by their EMPIAR ID.

Dataset	Type of protein	No. of labeled images	No. of labeled particles
10017	β-galactosidase	84	49391
10028	ribosome (80S)	300	26391
10081	transport	300	39352
10532	viral	300	87933
11056	transport	305	125908

**Table 3:** Comparison of performance of methods on different protein datasets Bold font denotes the best score of each metric.

	Blob detection			Template matching			*Topaz*			*CryoSegNet*			*CryoQSeg*		
Dataset	Precision	Recall	*F*_1_	Precision	Recall	*F*_1_	Precision	Recall	*F*_1_	Precision	Recall	*F*_1_	Precision	Recall	*F*_1_
10017	0.270	0.883	0.414	0.351	**0.970**	0.516	0.684	0.868	**0.765**	0.660	0.162	0.260	**0.769**	0.584	0.664
10028	0.281	0.888	0.427	0.177	0.898	0.296	0.669	0.900	0.768	**0.775**	0.817	0.796	0.739	**0.977**	**0.841**
10081	0.251	0.804	0.383	0.117	**0.968**	0.208	0.570	0.812	0.670	**0.672**	0.792	0.729	0.635	0.889	**0.741**
10532	0.121	0.033	0.051	0.341	**0.844**	**0.486**	0.347	0.096	0.150	0.402	0.140	0.208	**0.454**	0.341	0.389
11056	0.254	**0.849**	0.391	0.379	0.825	0.519	0.492	0.432	0.460	**0.633**	0.351	0.452	0.598	0.420	**0.493**

**Table 4:** Comparison of cryo-EM reconstruction performance for Crt-SPARTA-gRNA-tDNA oligomers (monomer, dimer, tetramer): published results (Gao *et al.*, 2024 ▸) versus *CryoQSeg*

	Published results			*CryoQSeg*		
Oligomers	Micrographs	Particles	Resolution (Å)	Micrographs	Particles	Resolution (Å)
Monomer	7080	1040721	3.27	2652	223045	3.39
Dimer	20299	614651	3.49	2652	12619	4.43
Tetramer	20299	1116433	3.41	2652	44565	4.30

**Table 5:** Particle picking performance of SAM-only versus HCT-assisted SAM on test datasets Bold font denotes the best score of each metric.

	SAM-only			HCT-assisted SAM		
Dataset	Precision	Recall	*F*_1_	Precision	Recall	*F*_1_
10017	0.756	0.409	0.531	**0.769**	**0.584**	**0.664**
10028	0.728	0.974	0.834	**0.739**	**0.977**	**0.841**
10081	0.623	0.834	0.714	**0.635**	**0.889**	**0.741**
10532	**0.457**	0.278	0.346	0.454	**0.341**	**0.389**
11056	0.562	0.257	0.353	**0.598**	**0.420**	**0.493**

**Table 6:** Particle picking performance of SFT alone versus SFT+RL on test datasets Bold font denotes the best score of each metric.

	SFT alone			SFT+RL		
Dataset	Precision	Recall	*F*_1_	Precision	Recall	*F*_1_
10017	0.769	0.554	0.644	0.769	**0.584**	**0.664**
10028	**0.748**	0.938	0.832	0.739	**0.977**	**0.841**
10081	**0.650**	0.828	0.728	0.635	**0.889**	**0.741**
10532	0.454	0.292	0.355	0.454	**0.341**	**0.389**
11056	**0.603**	0.410	0.488	0.598	**0.420**	**0.493**

**Table 7:** Summary of 3D reconstruction outcomes across three independent replicate runs using particles picked exclusively by *CryoQSeg* For each replicate, the numerical value corresponds to the final refined resolution in ångströms; the number of particles retained after refinement is listed within parentheses.

Dataset	Replicate 1, resolution (Å) (No. of particles)	Replicate 2, resolution (Å) (No. of particles)	Replicate 3, resolution (Å) (No. of particles)
10017	3.66 (30 391)	3.72 (22 927)	3.75 (31 337)
10028	3.91 (26 902)	3.99 (27 078)	4.01 (26 771)
10081	3.44 (21 424)	3.35 (34 033)	3.30 (32 688)
10532	3.04 (10 003)	3.16 (12 722)	2.95 (29 060)
11056	9.73 (18 861)	9.91 (22 764)	9.93 (21 090)
Crt (monomer)	3.39 (223 045)	3.29 (252 350)	3.37 (216 064)
Crt (dimer)	4.43 (12 619)	4.50 (22 694)	4.48 (13 835)
Crt (tetramer)	4.30 (44 565)	4.26 (30 247)	4.16 (32 434)

**Table 8:** Comparison of retained particle counts and final 3D reconstruction resolutions among five particle picking methods across five test datasets The optimal resolution for each dataset is shown in bold. *CryoSegNet* results are cited from its original publication; results of blob detection, template matching, *Topaz* and *CryoQSeg* are obtained via our in-house picking and refinement pipeline. Specifically, *CryoQSeg* adopts the results from the first independent replicate experiment.

	Blob		Template		*Topaz*		*CryoSegNet*		*CryoQSeg*	
Dataset	Particles	Resolution (Å)	Particles	Resolution (Å)	Particles	Resolution (Å)	Particles	Resolution (Å)	Particles	Resolution (Å)
10017	160 225	4.01	135 465	3.92	62 253	3.99	119 661	6.93	30 391	**3.66**
10028	83 460	4.07	133 884	4.11	35 500	4.09	47 764	**2.72**	26 902	3.91
10081	125 944	3.58	327 010	3.56	56 043	3.62	60 158	4.60	21 424	**3.44**
10532	23 691	7.73	217 464	3.62	24 212	3.77	67 219	4.13	10 003	**3.04**
11056	421 252	10.63	274 289	10.22	110 378	9.96	71 342	**7.88**	18 861	9.73

**Table 9:** comparison of performance of *UPicker*, *VMPicker* and *CryoQSeg* on different protein datasets Bold font denotes the best score of each metric within each row.

	*UPicker*			*VMPicker*			*CryoQSeg*		
Dataset	Precision	Recall	*F*_1_	Precision	Recall	*F*_1_	Precision	Recall	*F*_1_
10017	0.080	0.026	0.039	**0.795**	0.025	0.049	0.769	**0.584**	**0.664**
10028	0.059	0.036	0.045	0.593	0.116	0.194	**0.739**	**0.977**	**0.841**
10081	0.049	0.088	0.063	**0.660**	0.131	0.219	0.635	**0.889**	**0.741**
10532	0.107	0.063	0.079	0.364	0.015	0.029	**0.454**	**0.341**	**0.389**
11056	0.099	0.044	0.061	**0.710**	0.100	0.175	0.598	**0.420**	**0.493**

**Table 10:** Particle picking performance of different prompts on test datasets Prompt A: ‘generate segmentation mask for cryo-EM image’. Prompt B: ‘generate segmentation mask for cryo-EM image and mark the particle centers with red dots’. Bold font denotes the best score of each metric.

	Prompt A			Prompt B		
Dataset	Precision	Recall	*F*_1_	Precision	Recall	*F*_1_
10017	**0.769**	0.584	**0.664**	0.573	**0.748**	0.649
10028	0.739	**0.977**	**0.841**	**0.741**	0.869	0.800
10081	0.635	0.889	0.741	**0.669**	**0.931**	**0.779**
10532	0.454	**0.341**	**0.389**	**0.480**	0.285	0.357
11056	**0.598**	0.420	0.493	0.591	**0.602**	**0.597**

## Data Availability

The dataset for the study is available on https://github.com/BioinfoMachineLearning/cryoppp.

## Appendix A Training details

Training of the proposed framework is implemented in two sequential stages: supervised fine-tuning (SFT) and reinforcement learning (RL) fine-tuning. All experiments are conducted on 6 NVIDIA A100 GPUs with 80 GB memory per GPU, and the total training time is approximately 30 h. The AdamW optimizer is utilized for both training stages, which is the standard configuration for large vision model fine-tuning.

For the SFT stage, the model is optimized with an initial learning rate of 1 × 10^−4^ for 5 epochs. The LoRA rank is set to 32, and the dataset is repeated once. The Dice loss is integrated with a weight of 0.5, applied at every training step (probability = 1) and only activated when the diffusion timestep is below 1000. Additionally, a downsampling factor of 2 is adopted to accelerate training.

For the subsequent RL fine-tuning stage, a smaller initial learning rate of 1 × 10^−5^ is used to ensure training stability, with a total of 2 training epochs. The composite loss function adopts a reward weight of 1.0, a KL divergence weight of 0.01 and an SFT loss weight of 0.1. The diffusion timestep range for RL sampling is restricted to 200 to guarantee accurate reward estimation.

The 6-channel 80 GB A100 GPU cluster provides sufficient computational resources to support the training of the large vision model backbone and the entire two-stage fine-tuning pipeline.

## Appendix B 3D density maps reconstructed from picked particles

The precision, recall and *F*_1_ score of particle picking quantify a method’s ability to distinguish true particles from non-particles, yet these metrics do not directly reflect the quality of protein 3D density maps reconstructed from the picked particles – a key end product of primary concern to researchers. Reconstructing 3D density maps relies on complex algorithms that convert 2D particle images into 3D structures, and the performance of these algorithms depends on multiple critical factors: the fidelity of true particles, the diversity of particle orientations (to ensure comprehensive structural coverage) and the proportion of false-positive particles. These nuanced factors cannot be fully captured by standalone metrics such as the *F*_1_ score; thus, we further compared *CryoQSeg* with a suite of baseline picking methods including blob detection, template matching, *Topaz* and *CryoSegNet*, in terms of the resolution of 3D density maps reconstructed from picked particles on the CryoPPP test dataset.

To demonstrate the consistency and robustness of our picking pipeline, we performed three independent full reconstruction replicates using particles picked by *CryoQSeg*. For each replicate run, particle-position star files generated by *CryoQSeg* were imported into *CryoSPARC* for subsequent 3D *ab initio* reconstruction and homogeneous refinement. *Ab initio* reconstruction was carried out without any pre-defined starting structural model. During homogeneous refinement, higher-order aberrations, particle defocus, beam-tilt effects and spherical aberration as well as other optical artifacts were corrected. The reconstruction results from the three independent replicates are summarized in Table 7 ▸. Only minor resolution fluctuations are observed across replicates for every protein dataset, confirming that *CryoQSeg* produces stable particle picks that support robust downstream 3D reconstruction.

Table 8 ▸ summarizes the 3D density map reconstruction resolutions across five protein datasets, where we benchmark our *CryoQSeg* against multiple particle picking baselines. For blob detection, template matching and *Topaz*, we fully reproduced the complete particle picking and downstream 3D reconstruction pipeline in our laboratory to obtain reconstruction results; for *CryoSegNet*, we directly adopt the officially published reconstruction values reported in its original manuscript.

Across EMPIAR 10017, 10081 and 10532, *CryoQSeg* achieves the optimal 3D reconstruction resolution among all five evaluated picking pipelines. For EMPIAR 10017, *CryoQSeg* attains a resolution of 3.66 Å, surpassing blob detection (4.01 Å), template matching (3.92 Å), *Topaz* (3.99 Å) and the published *CryoSegNet* result (6.93 Å). Notably, *CryoQSeg* reaches this superior resolution while retaining far fewer particles than blob detection and template matching. Comparable performance advantages are observed on EMPIAR 10081 and EMPIAR 10532: *CryoQSeg* delivers resolutions of 3.44 Å and 3.04 Å, respectively, outperforming all competing baselines even with drastically smaller particle pools relative to template matching and blob detection outputs.

Nevertheless, *CryoSegNet* yields better reported resolutions on EMPIAR 10028 and EMPIAR 11056. For EMPIAR 10028, *CryoSegNet*’s published resolution is 2.72 Å; with a pixel size of 1.34 Å, the theoretical Nyquist resolution limit equals 2 × 1.34 = 2.68 Å. The reported value sits extremely close to this physical bound, casting uncertainty over the plausibility of this reconstruction outcome. To further validate the structural fidelity of our *CryoQSeg*-based reconstructions, we calculated the map-to-model FSC using *Phenix* for EMPIAR 10028 (PDB: 3j7a/3j79), yielding a resolution of 3.88 Å. This value is consistent with the gold-standard FSC resolution of 3.91 Å obtained from *CryoSPARC*, confirming the reliability of our reconstructions. On EMPIAR 11056, *CryoSegNet* achieves 7.88 Å, which outperforms *CryoQSeg*’s 9.73 Å, despite *CryoQSeg* extracting more candidate particles.

We observe massive disparities in particle counts generated by different picking algorithms. Blob detection and template matching frequently output hundreds of thousands of candidate particles, yet such oversized particle collections do not consistently yield superior reconstruction quality. Given these substantial cross-method discrepancies in retained particle statistics, differences in final reconstruction resolution cannot be solely attributed to particle picking quality. As such, these 3D reconstruction results serve as supplementary exploratory observations rather than rigorously controlled benchmark metrics.

## Appendix C Local-resolution analysis of reconstructed density maps

Global FSC-based resolution provides an overall figure-of-merit for a 3D reconstruction, yet it cannot characterize spatially heterogeneous map quality across different regions of the density map. Complementary local-resolution analysis quantifies voxel-wise resolution variations and reveals fine-grained differences among particle picking workflows. Since the original reconstructed density maps for *CryoSegNet* are not publicly accessible, local-resolution assessment could only be performed for maps generated from blob detection, template matching, *Topaz* and our *CryoQSeg*.

The local-resolution-colored density maps for the five EMPIAR test datasets are presented in Fig. 9 ▸. Lower ångström values (blue tones in color bars) correspond to superior local resolution, whereas higher values (yellow-to-red tones) indicate poorly resolved regions. On nearly all test datasets, reconstructions derived from *CryoQSeg* particle sets exhibit favorable local-resolution behavior, with larger fractions of the density map populated by high-resolution (blue-colored) voxels compared with competing picking baselines. For EMPIAR 10017, EMPIAR 10081 and EMPIAR 10532, the *CryoQSeg* density maps show visibly reduced fractions of poorly resolved red/yellow regions relative to blob detection, template matching and *Topaz*. Nevertheless, consistent with global resolution results, all four methods yield predominantly poor local resolution for EMPIAR 10028, and overall map quality remains limited for EMPIAR 11056 across all picking strategies.

## Appendix D Comparison with recent segmentation-based methods

We benchmark two latest segmentation-based particle picking algorithms, *UPicker* and *VMPicker*, against *CryoQSeg*. All training/test datasets and evaluation criteria are fully unified for fair comparison: the 22 EMPIAR CryoPPP training subsets are used for model training, and five held-out EMPIAR test datasets (10017, 10028, 10081, 10532, 11056) are adopted for validation, with a coordinate matching tolerance set to one-quarter of the particle box size. The two baselines adopt distinct architectures: *UPicker* uses a semi-supervised transformer, while *VMPicker* combines *CryoVMUNet* segmentation with SAM for particle box generation.

*UPicker* adopts a two-stage semi-supervised training pipeline. It first undergoes 50 epochs of A-LoG unsupervised pre-training on four RTX 3090 GPUs, followed by another 50 epochs of supervised fine-tuning with a unified box width of 240. During inference, the threshold sth = 0.25 is used to generate mask-filtered bounding boxes. *VMPicker* trains a *CryoVMUNet* backbone from scratch on a single RTX 3090 GPU for 200 epochs (batch size 8, lr = 0.001). Inference applies a SAM IoU threshold of 0.94 to filter candidate particle regions.

*UPicker* and *VMPicker* achieve strong performance in their original publications, yet official pre-trained weights and proprietary domain-specific tuning tricks are not publicly available. We reproduced both models strictly following their original paper descriptions and trained them from scratch on our unified dataset without access to internal optimization strategies. As shown in Table 9 ▸, self-trained *UPicker* and *VMPicker* exhibit substantially lower recall and *F*_1_ scores than *CryoQSeg* across all test datasets. This performance gap does not reflect inherent flaws of the two algorithms. The original authors’ high reported performance is likely enabled by proprietary pre-trained weights and unpublished training heuristics; full tuning resources would significantly improve their particle picking accuracy.

## Appendix E Ice contamination avoidance in particle picking

Benefiting from the powerful semantic representation and structural learning capabilities of visual large models, *CryoQSeg* exhibits exceptional immunity to ice contamination during particle picking. Notably, the model achieves precise particle localization without the need for explicit negative samples (*e.g.* ice artifacts, background noise) during training. By solely learning the intrinsic structural and morphological priors of target protein particles from positive samples, *CryoQSeg* can automatically distinguish genuine particles from ice contamination regions and non-particle backgrounds.

The qualitative particle picking results visualized in Fig. 10 ▸ validate this advantage across all six benchmark datasets, including five CryoPPP datasets and the Crt-SPARTA-gRNA-tDNA dataset. The red circles in each subfigure denote the particle coordinates identified by *CryoQSeg*, which clearly concentrate on valid protein particles and strictly avoid ice-contaminated regions. This intuitive visualization demonstrates that the proposed model effectively suppresses interference from ice artifacts, enabling robust and clean particle selection even in micrographs with severe ice pollution.

## Appendix F Exploration of training data annotation and prompt optimization for particle picking

We performed an exploratory study on training data refinement and prompt engineering for the visual large model toward cryo-EM particle picking. Specifically, we annotated red dots at the centers of protein particles in all segmentation masks of the training set and re-trained the visual large model using these annotated data. Concurrently, we revised the natural language prompt from the original ‘generate segmentation mask for cryo-EM image’ (denoted as prompt A) to ‘generate segmentation mask for cryo-EM image and mark the particle centers with red dots’ (denoted as prompt B). This design attempts to directly predict particle central coordinates via the predicted red dots, with the goal of bypassing the subsequent post-processing pipelines of SAM and Hough circle transform (HCT) for end-to-end particle localization.

The quantitative particle picking performance of the two prompt configurations is presented in Table 10 ▸, where we compare precision, recall and *F*_1_ score across five test datasets. On dataset 10017, prompt A achieves a higher precision (0.769 versus 0.573) and *F*_1_ score (0.664 versus 0.649), while prompt B yields a significantly improved recall (0.748 versus 0.584), revealing a clear trade-off between precision and particle recovery capability. On dataset 10028, prompt A outperforms prompt B in both recall (0.977 versus 0.869) and *F*_1_ score (0.841 versus 0.800), despite prompt B achieving a marginally higher precision (0.741 versus 0.739). In contrast, on dataset 10081, prompt B demonstrates comprehensive advantages: it increases precision from 0.635 to 0.669, recall from 0.889 to 0.931 and *F*_1_ score from 0.741 to 0.779, showing robust performance improvement across all metrics. For the low-signal dataset 10532, prompt A achieves better recall (0.341 versus 0.285) and *F*_1_ score (0.389 versus 0.357), while prompt B slightly boosts precision (0.480 versus 0.454) at the cost of reduced recall. On dataset 11056, prompt B enhances recall from 0.420 to 0.602 and *F*_1_ score from 0.493 to 0.597, with only a minor drop in precision (0.591 versus 0.598).

These results reveal that the modified training data and prompt strategy exhibits dataset-dependent performance: it delivers comprehensive improvements on datasets like 10081 and 11056 (characterized by densely distributed particles), but leads to performance degradation on datasets such as 10028 and 10532. This suggests that the effectiveness of particle center annotation and prompt optimization is closely tied to the structural characteristics of the target particles (*e.g.* density, signal-to-noise ratio), warranting further systematic investigation into the underlying mechanisms and optimal configuration strategies in future work. This exploration provides a valuable empirical reference for the development of end-to-end cryo-EM particle localization pipelines based on visual large models.
